# Supplementary material for: Improvement of the In Vitro Cytotoxic Effect on HT-29 Colon Cancer Cells by Combining 5-Fluorouacil and Fluphenazine with Green, Red or Brown Propolis
Source: Molecules. 2023 Apr 12;28(8):3393. doi: 10.3390/molecules28083393 (PMC10145548; doi:10.3390/molecules28083393)
Supplement: Supplementary file 1 [file molecules-28-03393-s001.zip › molecules-2259221-supplementary.pdf]

Supplementary material

Improvement of the In Vitro Cytotoxic Effect on HT-29 Colon Cancer Cells by Combining 5-Fluorouacil and Fluphenazine with Green, Red or Brown Propolis

Soraia I. Falcão [1,2,*], Diana Duarte [3,4], Moustapha Diallo [1,2], Joana Santos [3,4], Eduarda Ribeiro [3,4], Nuno Vale [3,4,5] and Miguel Vilas-Boas [1,2,*]

[1] Centro de Investigação de Montanha (CIMO), Instituto Politécnico de Bragança, Campus de Santa Apolónia, 5300-253 Bragança, Portugal
[2] Laboratório Associado para a Sustentabilidade e Tecnologia em Regiões de Montanha (SusTEC), Instituto Politécnico de Bragança, Campus de Santa Apolónia, 5300-253 Bragança, Portugal
[3] OncoPharma Research Group, Center for Health Technology and Services Research (CINTESIS), Rua Doutor Plácido da Costa, 4200-450 Porto, Portugal
[4] CINTESIS@RISE, Faculty of Medicine, University of Porto, Alameda Professor Hernâni Monteiro, 4200-319 Porto, Portugal
[5] Department of Community Medicine, Health Information and Decision (MEDCIDS), Faculty of Medicine, University of Porto, Alameda Professor Hernâni Monteiro, 4200-319 Porto, Portugal

*Correspondence: sfalcao@ipb.pt (S.I.F.); mvboas@ipb.pt (M.V.-B.); Tel.: +351-273303401 (S.I.F.); +351-273303309 (M.V.-B.).

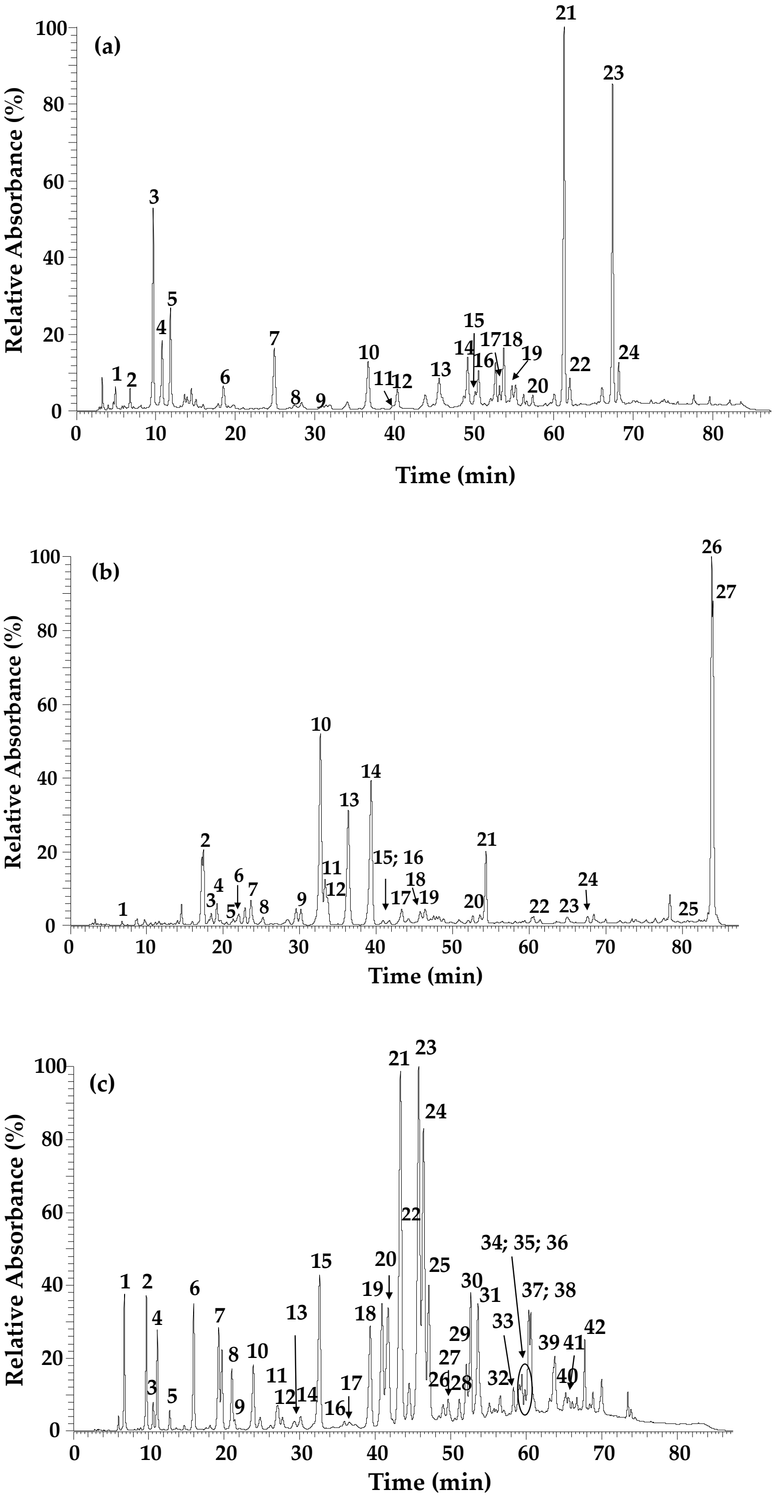

Figure S1. Chromatographic profile at 280 nm for the ethanolic extract: (a) green propolis; (b) red propolis; (c) brown propolis.
